# Molecular theranostics: principles, challenges and controversies

**DOI:** 10.1002/jmrs.836

**Published:** 2024-11-01

**Authors:** Geoffrey Currie

**Affiliations:** ^1^ Charles Sturt University Wagga Wagga New South Wales Australia

## Abstract

Theranostics is a new term for long‐established principles in nuclear medicine. The generalisability of the term means there is a very broad use of the term across the medical literature, not all of which is consistent with the intent in nuclear medicine. The term molecular theranostics better reflects the philosophy and application in nuclear medicine. Even with a clearer definition, there are a number of challenges or controversies whose debate provides a richer understanding of the principles and applications of molecular theranostics. Radioiodine imaging and therapy of hyperthyroidism and thyroid cancer provide the historical context for theranostics. The prototype molecular theranostic is the ^68^Ga/^177^Lu DOTATATE pair that targets somatostatin receptor subtype 2 in neuroendocrine tumors. The potential value of precision medicine of radiation dosimetry in molecular theranostics needs a balanced discussion with limitations of reactive dosimetry and the opportunities for predictive or pre‐treatment dosimetry. Despite challenges and limitations, molecular theranostics is a powerful tool in the precision medicine landscape. Molecular theranostics is a vehicle for improved outcomes in cancer patients with a future‐facing portfolio of opportunities.

## Graphical abstract

Molecular theranostics offers a powerful tool to drive precision medicine in nuclear oncology. While theranostics is not a new principle in nuclear medicine, recent advances in instrumentation and radiopharmacy have driven a reinvigoration and a broader suite of applications.

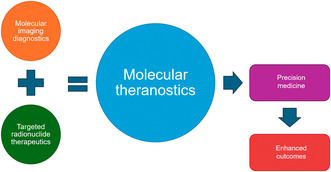

## Introduction

**Figure 1:**
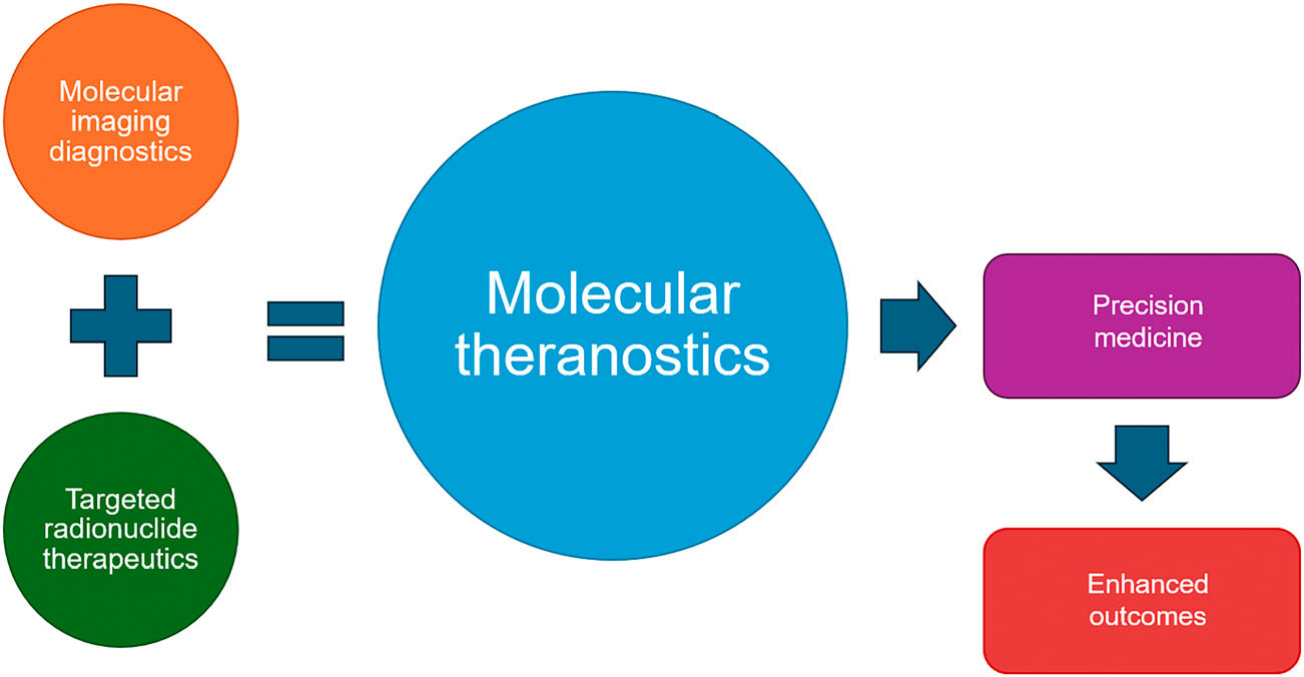
Schematic representation of theranostics in nuclear medicine and molecular imaging.

Theranostics is a relatively new term for principles adopted in nuclear medicine over a long period of time.^1^, ^2^, ^3^ As a portmanteau, the term theranostics combines parts of the words diagnostic and therapeutic to reflect the integration of diagnostics in shaping therapeutics. In the broadest sense, theranostics could be a pair of any diagnostic tool used to guide therapy but the term tends to be reserved for very specific pairs that behave in the same manner. For example, pairing a chest X‐ray with antibiotics for therapy of pneumonia could be classified as both image‐guided therapy and a theranostic pair but does not reflect the intent in treating cancer nor the philosophy of precision medicine. A narrower view might require the integration of a single molecule or entity for both diagnosis and treatment.^4^ Such an interpretation might include a wide variety of diagnostic (e.g. ultrasound, magnetic resonance imaging, fluorescence) and therapeutic (e.g. chemotherapy, radiotherapy, immunotherapy) pairs. In nuclear medicine, the term therapeutics is synonymous with the use of molecular imaging in positron emission tomography (PET) with targeted radionuclide therapies of the same molecular probe^1^, ^2^, ^3^, ^4^ (Fig. 1). To avoid confusion with other diagnostic and therapeutic pairs, the term molecular theranostics could be adopted.

### Theranostics or theragnostics?

Theranostics and theragnostics are words created by combining therapeutics and diagnostics. There has been debate about whether theranostic or theragnostic is the appropriate term, although they have tended to be used interchangeably. Recent efforts to clarify the issue used a linguistics expert who suggested theragnostics was imprecise but more consistent with the Greek origins of the words.^5^ While used to purport the appropriateness of theragnostic, it raises two important questions. Firstly, if neither is correct against the Greek origins then the least incorrect is not necessarily the appropriate choice; rather a third correct version is required (therapognostics). Secondly, a portmanteau is a literary tool with poetic and creative origins that is not meant to follow Greek or Latin language conventions. For example, “brunch” does not respect the linguistic origins of the word breakfast yet the portmanteau blends the two words neatly. Indeed, a portmanteau should blend fragments rather than splice root terms. According to ChatGPT, the conventions for creating a portmanteau include the combination of the beginning of one word and the end of another (e.g. theranostics), the first syllables of each word (e.g. theradiag), overlapping sounds (e.g. theragnostics), and deletion of redundant syllables (e.g. therostics) while maintaining pronounceability and meaning. Consequently, either approach is correct although theranostics is linguistically smoother and appears to have emerged as the preferred term in the literature. Nonetheless, theragnostic could be misunderstood as being agnostic to therapy.

## Molecular Theranostics

Molecular theranostics is the principle of pairing diagnostic and therapeutic radionuclides that share a common pharmaceutical or biomolecule.^1^, ^2^, ^3^, ^4^, ^6^ Consequently, the physiological behaviour of the radiopharmaceutical remains the same between diagnosis and therapy. In theory, only the physical properties of the radionuclide change function between diagnosis and therapy. A number of valuable theranostic pairs have emerged that have reinvigorated radionuclide therapies and reimagined the precision medicine landscape. The value of theranostic pairs comes from being able to provide more targeted cancer therapy,^6^ and might be more useful to target cancers not well managed with other therapeutic approaches or to provide a less aggressive approach for fragile patients.

**Figure 2:**
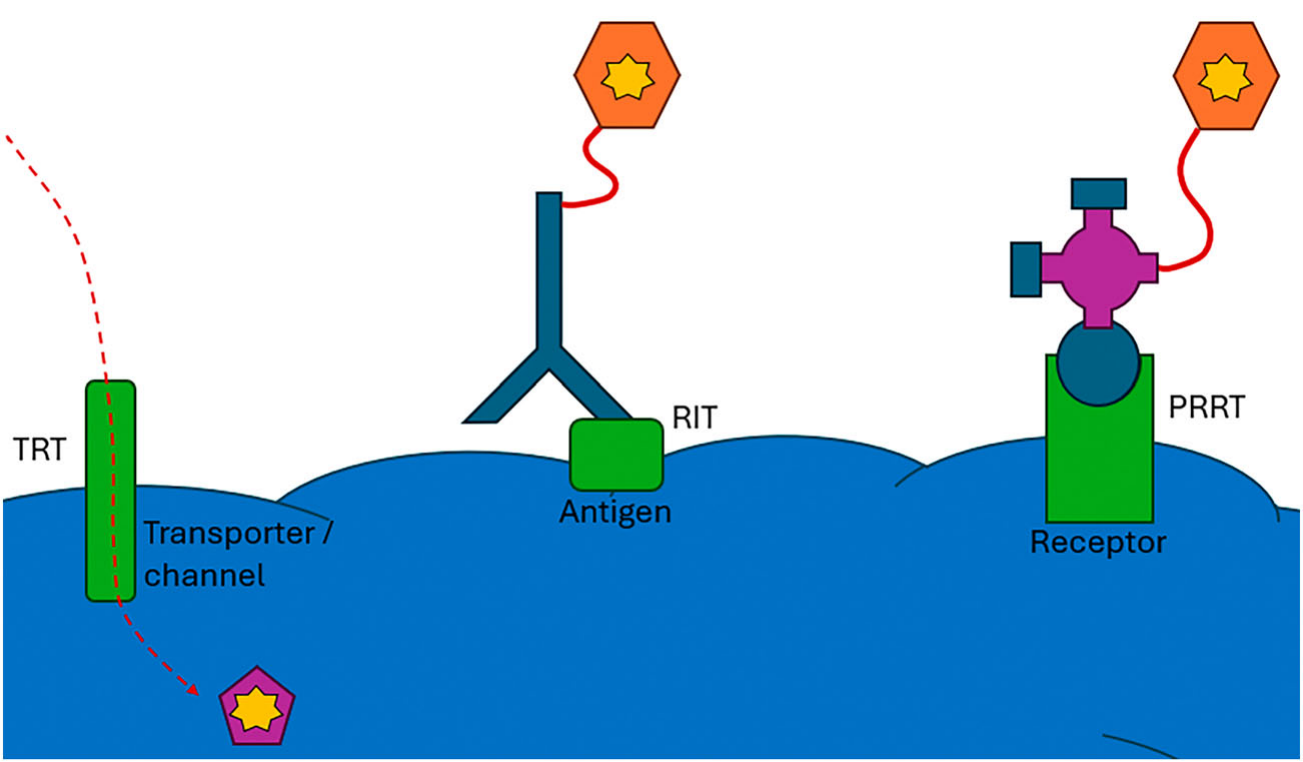
Schematic representation of molecular theranostics for TRT, RIT and PRRT. For TRT the chelated radionuclide enters the cancer cell via a transporter, channel or pump while for RIT the monoclonal antibody or fragment targets the cell surface antigen with the radionuclide chelated separately and joined by a linker. In PRRT, a cell surface receptor is targeted by the active portion of a small peptide with the radionuclide chelated and attached to the peptide via a linker.

Molecular theranostics aims to identify molecular targets in cancer tissues that can be exploited for optimised targeting of ligands for diagnostic imaging and therapy.^7^ A high degree of selectivity and affinity of ligands for cancer tissue targets, when radiolabelled with appropriate radionuclide pairs (diagnostic or therapeutic), provide accurate disease detection and delineation, staging, prediction of treatment response, determination of the appropriate treatment approach, and monitoring of response to therapy. Additionally, determining or predicting radiation dosimetry to target and non‐target tissues are also important goals.^6^ There are three general classifications of theranostic approaches (Fig. 2); targeted radionuclide therapy (TRT), radioimmunotherapy (RIT) and peptide receptor radionuclide therapy (PRRT).

**Figure 3:**
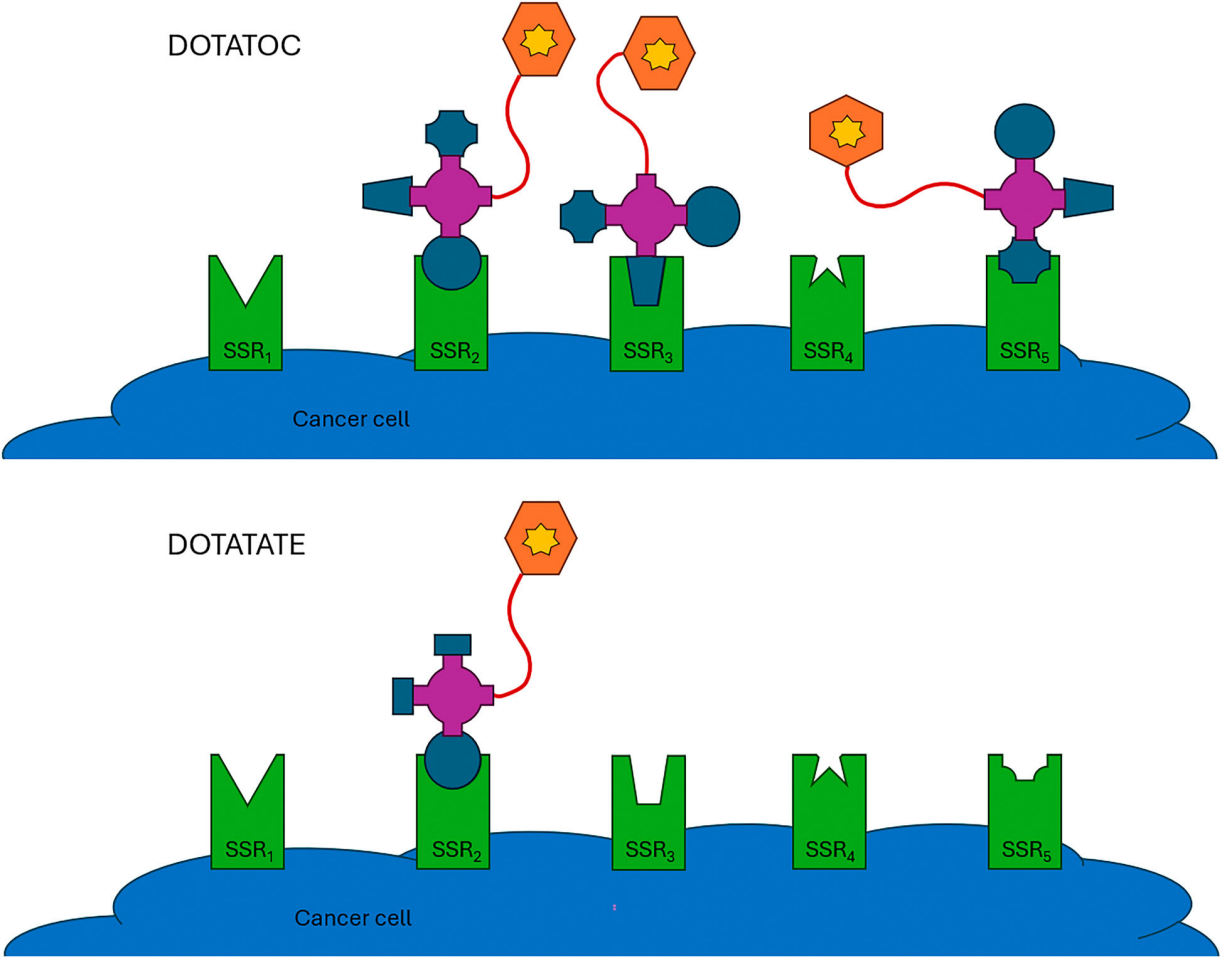
Comparison of the non‐selectivity for somatostatin receptor sub‐types by DOTATOC (top) compared to the subtype 2 selectivity of DOTATATE (bottom). The representation adopts the “lock and key” metaphor for the receptor (green) and active portion (blue) of the whole peptide (pink) attached via a linker (red) to the chelator (orange) where the radiometal (yellow) is bound. This allows non‐destructive labelling of the peptide to maintain molecular integrity.

The emergence of molecular theranostics has largely reflected developments in receptor targeting peptides for PET imaging and radionuclide therapy. The receptor principle relies on an overexpression of particular cell surface receptors or antigens that can be then targeted with a specific probe or tracer.^8^ Cell surface receptors and antigens respond to specific neurotransmitters, hormones, antigens, chemicals or substances (chemical ligand) that then trigger a response (signal) in the cell.^8^ Ligands can be selective for a specific receptor or non‐selective, can have a range of affinities for the receptors, can have a range of potencies of action at the receptor, and could be an agonist or antagonist. Receptors can be specific for a ligand or non‐specific, and can be inhibitory or stimulatory in action. For example, the ligand DOTATOC is partially selective because it has an affinity for somatostatin receptor sub‐types 2, 3 and 5 while DOTATATE is selective for sub‐type 2 (Fig. 3).^7^ DOTATATE also has a higher affinity for receptors (10‐fold over DOTATOC) and as a result, has higher localisation at the target receptors.^7^ Importantly, neuroendocrine tumours overexpress somatostatin receptor subtype 2 95% of the time.^7^ The somatostatin receptor is agonised by somatostatin to inhibit tumour cell proliferation, growth and angiogenesis which is the foundation of cold octreotide (peptide without a radionuclide) or hormone therapy, and which can be exploited with radionuclide pairs for DOTATATE. Conversely, prostate‐specific membrane antigen (PSMA) targeted ligands with high affinity for cell surface PSMA over‐expressed in prostate cancer cells, tend to be antagonists and, thus, block the action of the cell surface antigen. ^18^F‐FDG is an important part of image‐guided therapy but it is not considered a theranostic because, even if FDG were labelled with a therapy radionuclide, the non‐specific accumulation of FDG in normal tissues and non‐oncologic pathologies undermines targeted and precision therapies.

**Figure 4:**
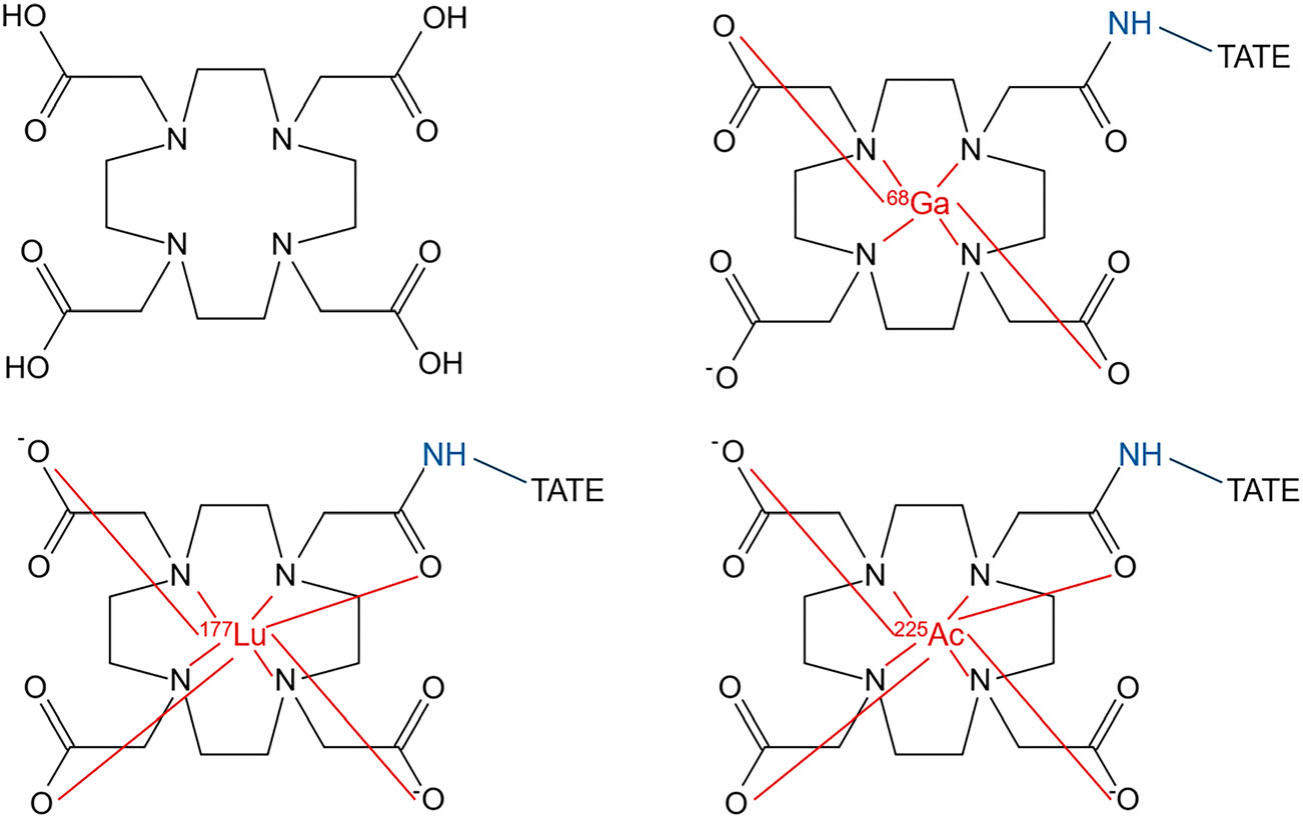
The DOTA chelator (left) with respective chelation of ^68^Ga (top right), ^177^Lu (bottom left) and ^225^Ac (bottom right) outlined in red with the peptide (TATE) connected to the chelator via a linker (blue).

In molecular theranostics, the radionuclide needs to be strongly bound to the peptide in a manner that does not alter the molecular behaviour of the peptide. Using radiometals (e.g., ^68^Ga) a chelator binds the radionuclide which is then attached to the peptide via a chemical linker^2^, ^6^ so that the overall peptide function (and specifically the active portion) is not interfered with. When the active portion of the peptide binds to the cell surface receptor or antigen, the radionuclide is co‐located to allow imaging or therapy. Dodecane tetraacetic acid (DOTA) is the most common chelator because it is used for ^68^Ga, ^177^Lu and ^225^Ac (Fig. 4) and it is the macrocyclic chelator that is used for gadolinium contrast in MRI. There are other macrocyclic chelators including nonane triacetic acid (NOTA) which is used for the aluminium‐fluoride (Al‐F) label, tetradecane tetraacetic acid (TETA) and sarcophagine (Sar) which is appropriate for ^64^Cu labelling. Additionally, there are a number of linear chelators including diethylenetriamine pentaacetic acid (DTPA) used by some gadolinium contrast agents, hydroxybenzylethylenediamine diacetic acid (HBED‐CC) used for PSMA‐11 and ethylene diamine tetramethylene acid (EDTA). The chelator binds the radiometal and connects to the peptide (targeting biomolecule) via the linker to allow radiolabelling without alteration to biological function and to minimise de‐chelation or de‐metalation, in both cases reducing radiation dose to non‐target tissues.

### What is a true theranostic pair?

The classic theranostic pair is ^123^I/^131^I in the radiochemical form (sodium iodide) for hyperthyroidism and thyroid cancer.^2^, ^3^ As sodium iodide, the diagnostic agent (^123^I) follows the same molecular pathway as the therapeutic agent (^131^I) and natural sodium iodide entering the thyroid via a symporter and then organification. This TRT approach might be considered a true theranostic pair. In hyperthyroidism, ^99m^Tc sodium pertechnetate is often substituted for the diagnostic agent because thyroid uptake adopts the same symporter mechanism. Unfortunately, ^99m^Tc sodium pertechnetate is then rapidly washed out of thyroid via the ATPase sodium/potassium pump, the mechanism that clears the sodium liberated from sodium iodide when the iodide is transported for organification. ^99m^Tc is not transported for organification and is, therefore, eliminated bound to the sodium. While ^99m^Tc sodium pertechnetate can be used to guide ^131^I sodium iodide therapy (image guided therapy) and the pairs share some molecular pathways, they are not true molecular theranostic pairs. Similar TRT approaches that are sometimes referred to as theranostic pairs but do not reflect the identical molecular pathway include the various agents for palliation of painful bone metastases. For example, ^99m^Tc diphosphonates, ^223^Ra chloride, ^18^F sodium fluoride and ^89^Sr chloride all localise in the hydroxyapatite crystal and reflect osteoblastic reactivity but ^99m^Tc diphosphonates localise as an analogue of inorganic phosphate via a type‐III Na/Pi cotransporter while ^223^Ra chloride and ^89^Sr chloride localise as calcium analogues via an annexin calcium channel, and ^18^F sodium fluoride localises via an anion exchanger.

**Figure 5:**
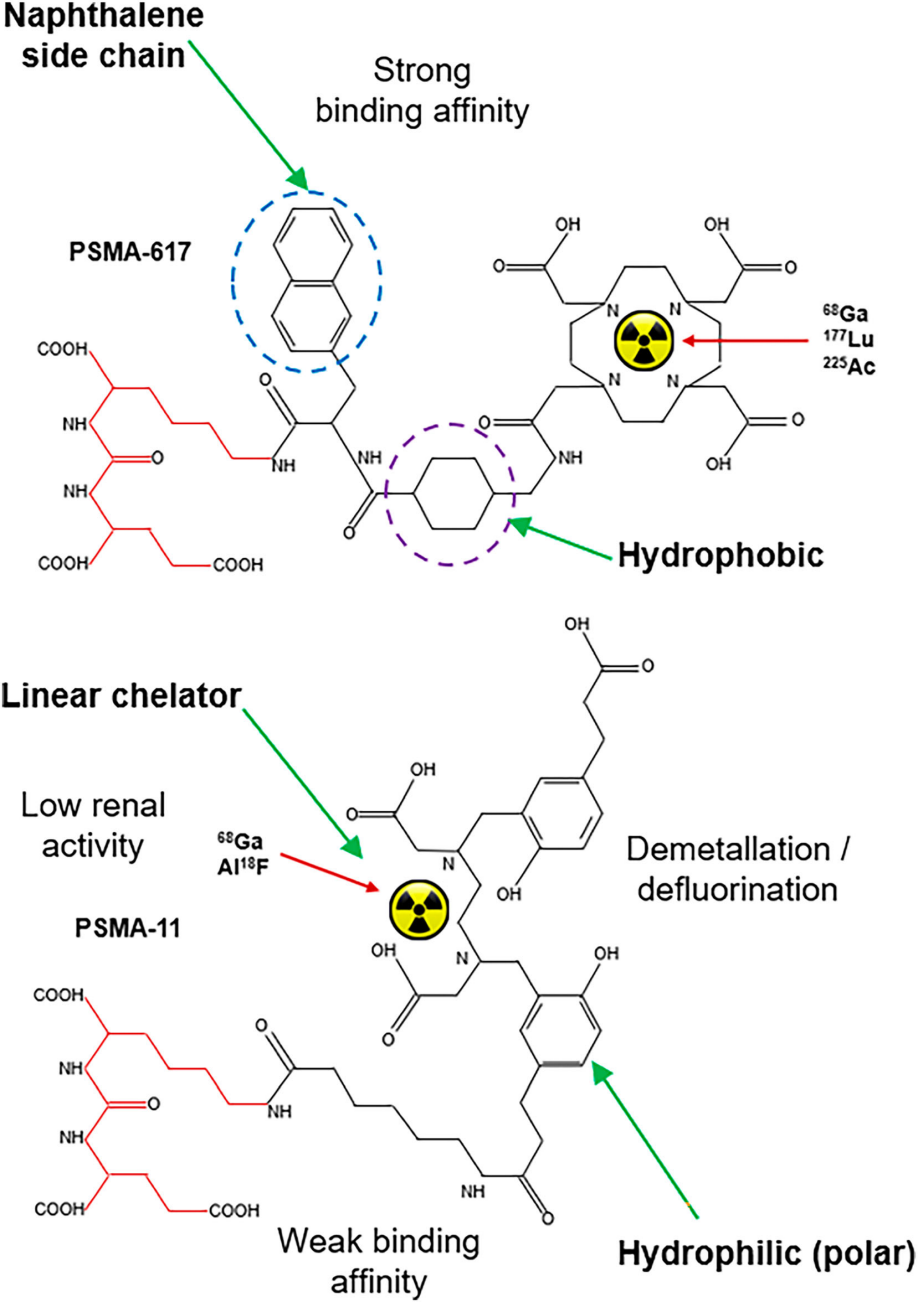
Schematic representation of PSMA targeted peptides showing a common active motif or pharmacophore that binds to the antigen (red). PSMA‐617 (top) is a macrocyclic (DOTA) chelator suitable for ^68^Ga, ^177^Lu and ^225^Ac chelation while PSMA‐11 (bottom) is a linear chelator (HBED‐CC) that accommodates ^68^Ga and the Al‐^18^F label. The naphthalene side chain and hydrophobic ring of PSMA‐617 increases binding affinity while PSMA‐11 not only lacks these accessory sites but the hydrophobic ring is replaced by a hydrophilic ring.

**Figure 6:**
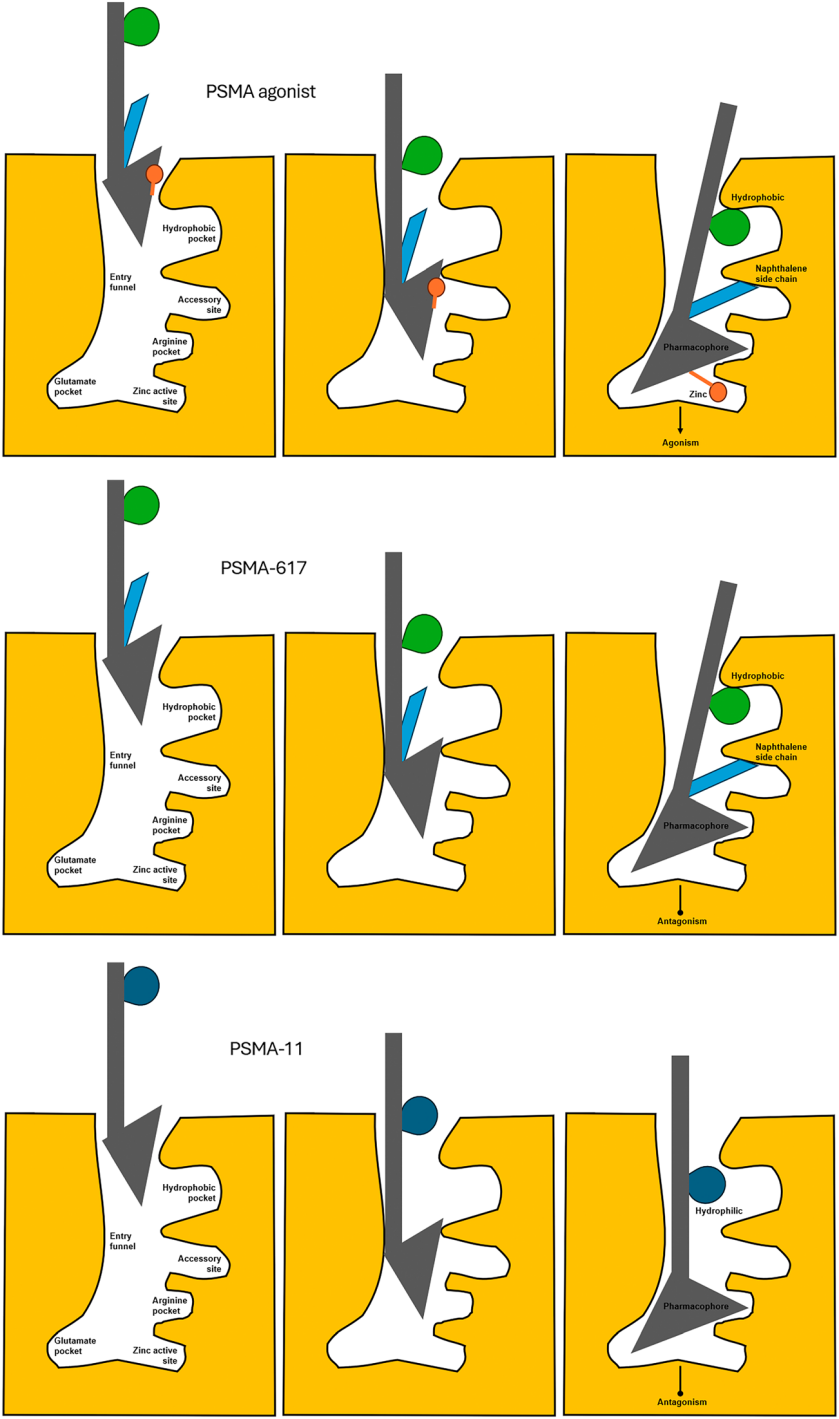
Schematic representation of the binding affinity of the endogenous PSMA agonist with the zinc finger to produce agonist activity (top). PSMA‐617 replicates the endogenous ligand with pharmacophore and accessory binding sites for high affinity and, in the absence of the zinc finger, antagonist activity (middle). PSMA‐11 lacks accessory binding sites and has incompatibility with the hydrophobic pocket for lower affinity binding (bottom).

For PRRT, the same principles apply. The prototype theranostic pair is ^68^Ga/^177^Lu DOTATATE. For DOTATATE, the chelator is DOTA and the peptide is TATE regardless of the radionuclide. This is a true theranostic pair. ^64^Cu SarTATE is the same peptide but different chelator (DOTA for ^177^Lu and a sarcophagine for ^64^Cu) which produces slightly different biodistribution and might be considered on the fringe of meeting the definition of a true theranostic. For PSMA targeted theranostics, while the pharmacophore or motif remains identical across probes (ie. the active portion of the peptide), there are considerable differences in chelators and peptide structure for different versions. For example, PSMA‐617 has antigen binding with two additional accessory pockets (high affinity) and a DOTA chelator while PSMA‐11 does not have the accessory binding sites (lower affinity) and uses a linear chelator (HBED‐CC) which is more susceptible to demetallation (unbound radiometal) (Fig. 5). In this scenario, ^68^Ga‐PSMA‐11 and ^177^Lu‐PSMA‐617 are not true theranostic pairs (Fig. 6). Furthermore, ^18^F‐PSMA‐11 has the additional variation of using a secondary binding to aluminium (Al‐F) to create a pseudo‐radiometal chelation which has an additional array of risks for demetallation (e.g. serum aluminium levels). While this classification may have limited impact clinically today, it is inconsistent with the underlying principle of precision medicine and will undermine the accuracy of future applications of predictive radiation dosimetry. Nonetheless, the ready availability of cyclotron‐produced ^18^F‐PSMA‐11 combined with cost and availability challenges of ^68^Ga generators with onsite ^68^Ga‐PSMA 11 synthesis, are important considerations. That is, ^18^F is widely and abundantly available and readily transported with a 110 minute half‐life which creates flexibility in scheduling and patient volumes. ^68^Ga generators are a more scarce resource with increasing demand putting pressure on supply. Furthermore, a generator is dose‐limited for a particular day which limits the number of patients that can be imaged. More recently, cyclotron production of ^68^Ga has been investigated.^9^ Until the benefits of predictive AI‐driven radiation dosimetry are clinically realised, PSMA‐11 is a suitable pair for PSMA‐617.

The challenge in molecular theranostics is to vary the physical properties of the radiometal/radionuclide (emission type, energy and half‐life) to allow either imaging or therapy (or both) while matching the chemical and biological properties of the probes.^10^ For example, ^68^Ga and ^177^Lu share some important chemical properties that allow use of the same chelator (DOTA) and peptide (TATE) but there are subtle differences in chemical structure (Fig. 4) that can produce variations in biological behaviour. Most commonly these are negligible but certainly time (longer half‐life of ^177^Lu) plays a factor. These issues are subtle and do not warrant concern clinically but is sufficient justification to explore theranostic pairs of the same element in the model of ^123^I/^131^I. Potential examples include ^86^Y/^90^Y and ^44^Sc/^47^Sc which, if suitable for ligand labelling, would produce authentic theranostic pairs.

## Radiation Dosimetry

There is a benefit in molecular theranostics to being able to measure radiation dosimetry to target and non‐target tissues so radiation dose burden can be calculated.^6^, ^8^ For most therapeutic radionuclides, imaging after therapy is not practical (e.g. ^90^Y). While there is evidence of some value associated with Bremsstrahlung imaging, PET imaging following pair production and Cerenkov luminescence,^11^ image quality is poor and dosimetry calculations limited as a result. Bremsstrahlung radiation are photons produced by the rapid slowing of higher energy beta particles and while they have no discrete energy, gamma camera imaging has been undertaken.^11^ A more discrete energy (511 keV) is produced by low abundance pair production associated with beta decay but background noise hinders image quality.^11^ Cerenkov radiation occurs when a particle moves through matter faster than the speed of light in the same matter but relies on the light (bioluminescence) being detectable outside the patient.^11^ Some therapeutic radionuclides have both particulate and photon emissions with sufficient abundance and appropriate energy for imaging. For example, ^177^Lu allows gamma imaging with low abundance gamma emissions at 113 keV and 208 keV. These gamma emissions allow sequential whole‐body imaging of therapy patients to determine biodistribution over 7–10 days with measurement of tissue dose and calculation of total dose burden. This is useful for evaluating tumour dose burden or that of a specific critical organ (e.g. kidney) that may be at risk of toxicity. There is also the chance to use this information to personalise subsequent doses to maximise target tissue dose and minimise collateral damage.

Ideally, pre‐therapy imaging would provide a map of biodistribution to determine suitability of theranostic treatment approaches and allow determination of radiation dosimetry to tumour and critical organs (non‐target organs). This would allow optimisation of the therapy dose based on the individual patient circumstances; precision medicine. The limitation of molecular theranostics is that the typically short half‐life of diagnostic radionuclides means the biodistribution reflects the period immediately post injection of the tracer and may be less indicative of how that biodistribution will vary over subsequent hours and days. There are a number of solutions for this problem. The first is the emergence of total‐body PET systems that provide substantially higher count rates (sensitivity/efficiency) and may allow delayed imaging of ^68^Ga probes at 12 and 24 h. These images may be more reflective of the longer duration biodistribution of the therapeutic probe. The second approach is the emergence of long‐lived imaging radionuclides like ^64^Cu (12.7 h) and ^89^Zr (78.4 h) whose long half‐life would permit delayed imaging. The limitation is that the longer half‐life also negatively influences radiation dosimetry. In both these approaches, at the very least, they would provide additional data for an artificial intelligence solution. The third option, therefore, is the use of deep learning algorithms to predict dosimetry of the therapy radionuclide from the diagnostic images.

### Artificial intelligence

**Figure 7:**
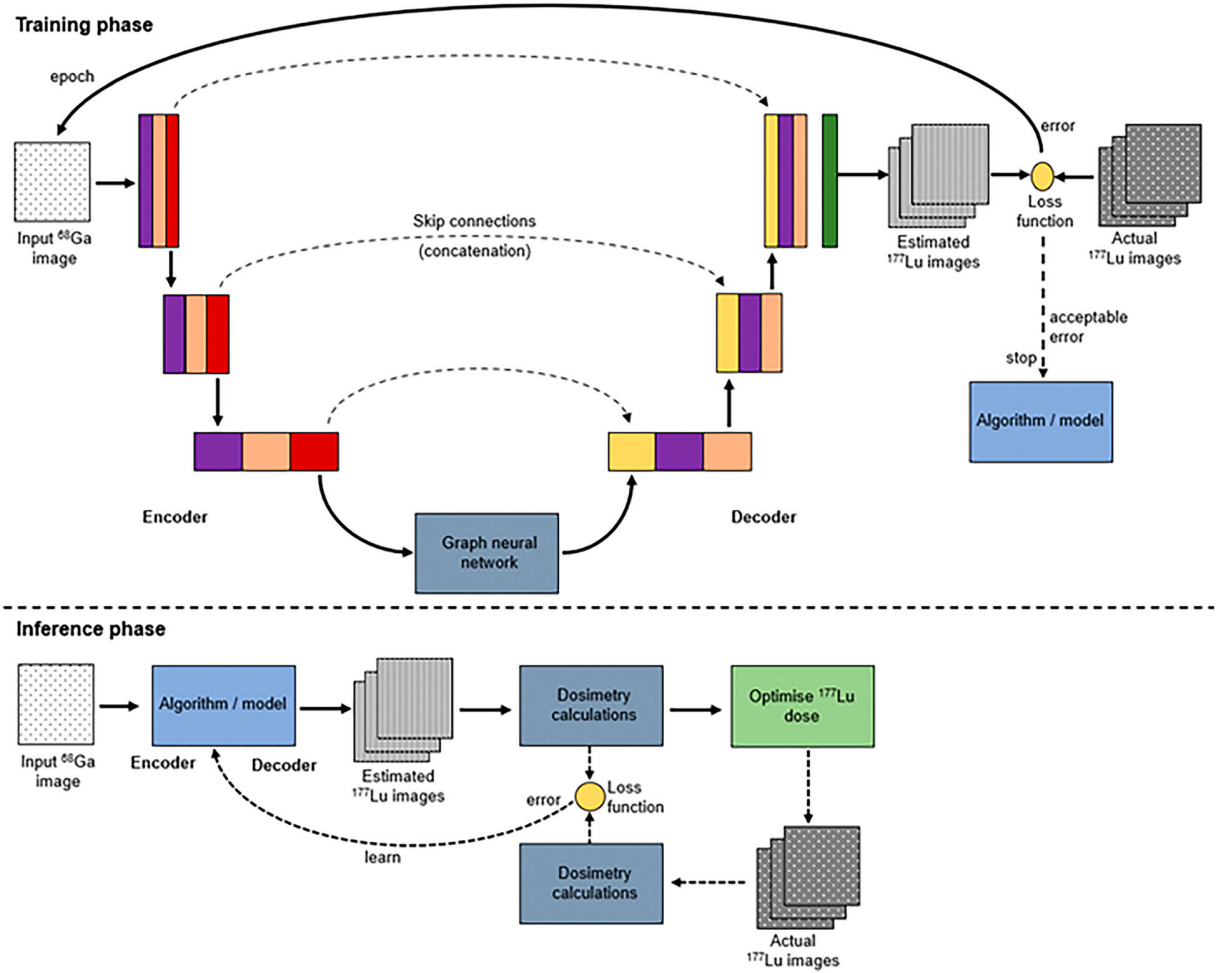
Schematic representation of an encoder‐decoder GNN in the U‐net architecture that could be used to develop dosimetry‐based optimisation of therapy doses of ^177^Lu based on the ^68^Ga‐PET/CT (reprinted with permission^12^).

The emergence of some of the deep learning architectures could provide solutions for predictive dosimetry from the diagnostic imaging.^10^ The vast majority of deep learning algorithms in nuclear oncology have been tailored for lesion detection, stratification, classification or segmentation and radiomic feature extraction.^10^ The role of artificial intelligence in radiation dosimetry in theranostics remains an exciting prospect and priority.^10^ Indeed, the capacity for predictive radiation dosimetry of the therapeutic dose from the initial diagnostic imaging would provide authentic molecular theranostics and drive precision medicine. For example, using the ^68^Ga‐PET/CT image to predict ^177^Lu therapy probe biodistribution could allow prediction of tumour dose burden and non‐target tissue dose burden (before administration of the therapy dose) which could allow customisation and optimisation of the patient dose. An encoder‐decoder U‐net with graph neural network or similar convolutional architecture could achieve this if it were trained to predict therapy dosimetry against a large dataset of original ^68^Ga‐PET scans and the corresponding serial ^177^Lu gamma‐based dosimetry. This would allow predictive dosimetry and personalised therapy doses based on optimising tumour burden and minimising non‐target tissue dose and toxicity risk (Fig. 7). The algorithm could continue to learn because the ^177^Lu‐based follow‐up images could be used to provide the grounded truth for each subsequent patient and adjust the network where deviations are noted.

## Conclusion

Despite challenges and limitations, molecular theranostics is a powerful tool in the precision medicine landscape. Molecular theranostics is a vehicle for improved outcomes in cancer patients with a future‐facing portfolio of opportunity. Emerging approaches include innovations in radionuclides and ligands that will continue the transformation of clinical practice.

## Funding

The authors declare that no funds, grants, or other support were received during the preparation of this manuscript.

## Conflict of Interest

The authors declare no conflict of interest.

## Ethics Approval

Human Ethics and Consent to Participate declarations: not applicable.

## Data Availability

Data sharing is not applicable to this article as no new data were created or analyzed in this study.
